# Dimensions of operational stress and forms of unacceptable risk taking with small arms and munitions

**DOI:** 10.1186/s40696-015-0001-4

**Published:** 2015-05-11

**Authors:** Uzi Ben-Shalom

**Affiliations:** Department of Sociology and Anthropology, Ariel University, Ariel, 40700 Israel

**Keywords:** Operational stress, Risk-taking, Safety climate, Small arms and munitions, Personality profile

## Abstract

**Background:**

Accidents with small arms and munitions during deployment is a significant safety concern for leaders and safety specialists in combat units. Operational stress may lead to forms of unacceptable risk taking with small arms that may underlie some of these accidents. The present research studied the correlation between two dimensions of operational stress, two forms of risk taking with small arms among combat unit soldiers and possible mediators. The dimensions of operational threat, negative affect and personality profile from the EPQ-R-S were predictors; "exaggerated preparedness" and "risky games with small arms and munitions" were dependent variables; safety climate of the platoon served as a mediator variable. The participants were 461 compulsory service combat soldiers in 31 companies. This field study was conducted during period of top security alert.

**Results:**

The results reveal that perceived threat is indeed correlated with exaggerated operational preparedness whereas general emotional state was correlated with risky games with small arms. Safety climate mediated only the correlation between general emotional state and risky games with small arms and munitions. Preparedness and risky games were predicted by the interaction of Psychoticism and the Lie Scale from the EPQ-R-S.

**Conclusions:**

The results may enhance the efforts in reducing risk taking and prevention of accidents with small arms and munitions during and following deployment.

## Background

### Operational stress and unacceptable risk taking

Military operations in the last decades entail far more diverse challenges than those described in the psychological and psychiatric literature developed following world war two. Often these challenges are described as operational stress [1]. Two significant components in this composite of challenges are injury and death from hostile enemy actions with the negative affect derived from prolonged boredom, lack of action and monotony [2]. Yet, such an exhausting routine can suddenly and without control be interrupted by an enemy attack [3]. Typical situations are sudden attacks on military posts, skirmishes during patrols, mortar fire attacks and explosion of IED’s. Research provides evidence proving that exposure to operational stress especially exposure to horrific events can lead to excess risk taking following military service [4]. The majority of literature regarding the correlation between operational stress and risk taking deals with risky behaviors after the operation activities including reckless driving, alcohol intake, and even a false feeling of ‘post-combat invincibility’. Only number of research did examine risky behaviors characterizing the operational action in theater as dangerous operational driving or games with small arms and munitions. Games with small arms or munitions are risk taking behaviors hardily reviewed in the literature, although it might very well be correlated with the abovementioned false feeling of invincibility [5].

### Unacceptable risk taking with small arms and munitions

Excessive or ineligible use of fire arms is a significant social problem [6] occurring with illegal bearing of arms in schools, usually associated with delinquency or rites of passage. This behavior is affiliated to drug abuse and other criminal behaviors or as an expression of suicidal and depressive behavior. The covert aspect of these risky behaviors places difficulties for field study thus relying on the wide use of data from inquiry reports of accidents or legal information [7]. As consequence the characteristics of many of these dangerous games with arms remains unclear. The present research studies the subject from another aspect, while examining defined types of risk taking behaviors and factors possibly leading to these behaviors.

Unacceptable risk taking with fire arms and munitions in the military can be defined as unauthorized use or handling of such equipment [8]. These are usually rare behaviors within the army leading to a few cases of accidents compared to other risks as road accidents [9]. Nonetheless, the disturbing nature of this safety risk requires allocation of substantial resources to prevent this type of risky behaviors. Military action essentially involves risks, yet it is important to differentiate between a risk that is part of the military mission and therefore inevitable and risk that is not part of the mission and is unacceptable [10]. An emphasis should be placed on the fact that not all of the games with small arms on the same level of risk: It is useful to differentiate between’theatrical’ games with small arms (as games of roulette or aiming the small arms at a person) or 'technical' games (as dissembling and assembling of a fire arm). No detailed definition exists regarding types of games with small arms in the military context. In this study the definition of unacceptable risk taking with fire arms or munitions has been broaden by examining this subject in operational setting.

### Firearms and coping with operational stress

Operational action can lead to various feeling including attentiveness and a sense of goal and purpose as well as anxiety and fears. On the other hand, operational action can involve high tempo actions accompanied with feelings of threat and danger. However operational actions are often monotonic and are characterized by lack of stimulation, boredom and fatigue. There are contentions that continuous operational action can create a bad influence on mental well-being [11]. This study examined the correlations between feelings of threat and boredom in operational setting with risk taking behaviors with small arms and munitions. Glickson, Ben-Shalom and Lazar (2004) have recently found correlation between dimensions of personality and willing to take uncalculated risks with small arms [12]. In their research they used the Eysenck Personality Questionnaire – Revised - Short Form (EPQ-R-S). Their findings demonstrate that the dimension of psychoticism (P) one of the three dimensions in Eysenck’s theory of personality (together with extraversion and neuroticism) is positively correlated with willingness for risky games with small arms. A personality profile with a high score on this dimension is correlated with limited impulse control, aggressiveness, hostile interpersonal relations and risk taking. However the previous study was conducted in a period of relative low security alert, several years before the outbreak of the second ‘Intifada’. This period was characterized with lack of significant operational activity or combat. One of the conclusions from this preliminary research was that this topic requires continued investigation in situations of higher operational stress. On the EPQ-R-S scaled used in the study included a dimension Lie = L intended for monitoring the style of the respondents answer. This dimension can be used to create a personality profiles which I can assume will tend to report risky behaviors.

I emphasized that operational stress contains opposing aspects of threat and boredom or a combination of the two. I claim each of these fundamentals has a unique influence and separate affinity to ways of coping. Threat and anxiety are correlated with the fight-flight mechanism while boredom to hedonistic sensation and thrill seeking. There are a variety of ways for coping with operational stress. Some can be 'healthy' (as humor) than other ‘less healthy’ (taking uncalculated risks). A situation of threat during deployment can lead to changing the way the personal rifle and munitions are being handled, especially concerning the level of readiness. An exaggerated expression of operational preparedness can be moving around while there is a round in the chamber or with loaded magazine when the situation doesn’t warrant it. This kind of behavior is more common at certain hours as at night time, when the soldier is alone or isolated, especially after an enemy attack on the post or station. The expression describing this situation is the hypothesis of ‘threat- preparedness'. Two accident reports from the year 2001 represent these cases the first during an infantry patrol and the other while being at a highly threatened post frequently invaded by infiltrators.

“The soldier was injured due to a bullet discharged from his personal rifle after he tripped and fell from a terrace … he was walking with a round in the chamber in violation of the orders. When he stood up and placed his hand on the trigger lock and unintentionally pulled the trigger. As consequence he suffered minor injuries to his leg.”

"The soldier, a sergeant in rank was injured when a shock grenade exploded in his palm while he was improvising a trap using barbed wire tied to the pin… he was attempting to make a means to alert the post from infiltration.”

Accidents caused by taking unnecessary risks with arms occur since there aren’t any good protocols for monitoring the state of the arms with high risk for human error due to fatigue, forgetfulness or distraction. There are reports in Israel of various accident with personal arms possibly correlated with these situations, in the house or in living quarters in the guard posts and army forward areas. In addition this can also lead to a discharge of a bullet while cleaning the small arms or during routine technical handling. Literature dealing with accidents mentions errors and faults due to the wrong order of actions when the operator of the technical equipment changes the protocol for handling the equipment in order to compensate on technical faults. These actions are usually characteristic of highly motivated and over competent operators. They were described in the past as ‘compensatory behavior’ [13]. These behaviors are highly influenced by common norms in the operator’s organizational environment or safety climate in the unit. Research conducted also in the I.D.F. demonstrated that safety climate is negatively correlated with accidents in combat units [14]. However, these studies did not examine the behaviors of risks taking with arms and the affinity between this behavior and the safety climate. In addition such studies were not conducted during emergency or high operational stress.

Operational stress can affect the general emotional state not only arousing fears or feeling of threat. Operational during deployment may often be characterized by continuous, prolonged, monotonic action. Negative affect under such circumstances arouse by the combination of boredom, alienation, isolation and fatigue together with delayed gratifications. In this situation, unauthorized games with arms and munitions -mainly with pyrotechnic devices as fireworks and firecrackers - are intended to ease the boredom and create thrills. This behavior is likely to be common among a certain type of personality profile, those who enjoy thrills and have low self-control. Individuals with this personality profile dislike states of boredom and low stimulation [15]. The correlation between these variables was reported in the literature. However, the majority of the research was conducted among soldiers after returning from their deployment tours overseas. While the current research study was conducted during the operational action itself, in the unit’s stations and posts and in diverse real life situations. This research focused on the soldier general emotional states. The correlation between these emotions and games with arms was name hypothesis of ‘negative affect-dangerous games.’ In recent years, reports on number of these types of accidents occurred during routine operational action. The following quote demonstrates a similar case:

“Three soldiers from the response squad were resting in a structure within a settlement. Corporal ‘A’ was playing with the deceased rifle and shot him in the face…during the week prior to the accident they both played with their rifles aiming them at each other” (Ben Shalom & Rothstein, 2001:30).

The first research hypothesis was that I will find positive correlation between perception of the operational threat and behaviors of exaggerated operational preparedness. The second hypothesis was that I will find positive correlation between negative affect and playing dangerous games with small arms and munitions. The first hypothesis was named ‘threat-preparedness’ and the second ‘negative affect-dangerous games’. The basic assumption was that a high level of perceived operational threat would lead to high operational preparedness. Another basic assumption was that negative affect can of course derive from many sources; it will lead to dangerous games when it results from lack of stimulations. In this situation a dangerous game can seem as fun or excitement. This assumption is mainly relevant for the group of participants with a high level of Psychoticism-P. This personality dimension has a positive correlation to low self-control, risk seeking and aggressiveness. Individuals who have a tendency to seek thrills also despise boredom and need a higher amount of stimulations.

Both hypotheses are based on different psychological mechanisms: the first is the arousal response of ‘fight-flight’ created by fear the second is ‘impulsive-hedonistic’ risk taking created by boredom [16]. The two mechanisms are independent; therefore they can be separate variables explaining very different risky behaviors. Empirical observation of these assumptions can contribute to our understanding of risk taking behaviors with arms or munitions, which as mentioned above are extremely difficult to examine in real-time conditions. Comprehension of the influence of these mechanisms can help reinforce safety regulations during deployment as well as activities when on leave.

The third hypothesis refers to the influence of safety climate on risky behaviors. I assume that safety climate of the unit is an influential factor for decreasing risky behaviors. Although, it is still unclear how the safety climate is correlated with risk taking with small arms and its affinity to operational stress. Therefore I assumed that safety climate in the unit is a mediator variable. When the unit safety climate is high, then a high level of perceived threat or negative affect will not be correlated with exaggerated preparedness or dangerous games.

The fourth hypothesis is based on previous research demonstrating that the personality dimension of P has a positive correlation with risk taking with arms. In order to test this hypothesis I combined the variables from the EPQ-R-S questionnaire. I assumed that high P profile and low L profile is the profile which will report a high tendency for dangerous games with arms. This hypothesis was based on our previous study as well as on other research, demonstrating that these dimensions have a capacity of predicting risky behaviors in the army and among juvenile delinquency [17,18].

## Methods

### Participants

The participants were 461 soldiers in compulsory service assigned to 31 companies in the ground forces. The majority (72%) were infantry and paratroopers, the armored corps (22%) and artillery (5%). The majority of the participants were men (96%). Among the preliminary sample 4% were officers. For data analysis the officers and women were excluded due to various rank and gender differences concerning illegal use of arms [9]. Therefore the final sample was 420 participants. The mean age was 20.1 (SD = 0.95; age range 18–24) and mean duration of service 19.8 months (SD = 9.36; range 6–42).

### Methods

The data was collected using anonymous questionnaires delivered by three research assistants in military posts. The participants were asked to assist in the research and it was explained that this activity was not obligatory. Questionnaire included questions about taking risks, operational preparedness and the safety climate in the unit. Finally, they completed personality questionnaire and background details.

#### Perceived treat

Perceived operational threat was evaluated with two separate measures: the frequency of different threats in the operational arena and exposure to death and injury due to hostile activity. The first variable included 8 questions examining the frequency of threats as mortar fire, IED explosions and shooting. The scale of the answers was between 0 (never) to 4 (everyday). The internal validity of this scale was good (α = 0.80). The second measure examining exposure to injuries included two questions regarding exposure to death and injuries. 28% of the participants claimed there were injured peers from their unit and 7% claimed they personally were exposed. These questions were combined into one measure in which 1 = no exposure to the injuries or death (n = 283, 68%); 2 = injuries and death in the unit (n = 91, 21%); 3 = personal exposure to the injuries or death (n = 30, 7%). There was moderate correlation between these two questions (r = 0.38, p < 0.001) therefore they were standardized and combined to one measure. This measure represented the participant’s perception of operational threat.

#### General emotional state (GES)

the general emotional state of the participant was evaluated by examining positive and negative affect. Although the research hypotheses focused on negative affect, the positive affect cannot be dismissed in order to achieve a more precise estimate in this issue. The participants answered a questionnaire with 16 questions examining the frequency of various emotions they felt during operational action they recently participated. This questionnaire was a version of the Affectometer 2 questionnaire [19] specially adapted for life in a combat unit during deployment. The questions were very short examining the frequency of 8 positive and 8 negative emotions during the past two weeks of operational action. (Examples of questions for the negative dimension were: ‘fatigued’, ‘exhausted’, ‘hopeless’; for the positive dimension were: ‘vigorous’, ‘happy’, ‘hopeful’). The scale ranged from 1 (seldom) to 5 (very often). Principal component Varimax revealed three factors with high inner reliability (Cronbach's alpha ranged between 0.74-0.86) described as: fatigue (items as: ‘fatigued’, ‘exhausted’) vigorous (items as: ‘energized’, ‘excited’) and worried (items as: ‘worried’, ‘helpless’). The correlation between the components was high (Pearson correlation between 0.56-0.46). Later a new variable was created for a general emotional state by subtracting the negative variable from the positive variable. Specifically: the higher the measure the stronger the positive affect. This calculation allows taking into consideration the positive and negative aspects of the emotional state.

#### Exaggerated operational preparedness and dangerous games

Exaggerated operational preparedness was evaluated in a questionnaire presenting 5 forbidden behaviors of exaggerated operational preparedness and questioning the degree they characterized the soldiers in the unit. The scale ranged between 1 (never) to 5 (very often). Examples of these questions included: “Soldiers in the post walk with a bullet in the chamber to be prepared for an attack on the post”; Soldiers prepare explosive traps to deter infiltration into the post”. The inner reliability correlation was high (Cronbach's alpha was 0.80). Forbidden games with arms and munitions were evaluated by a questionnaire presenting 5 forbidden behaviors characterize the soldiers in the unit. The scale ranged between 1 (never) and 5 (very often). Examples of these questions included “Soldiers aim their rifles towards each other as a game”; Soldiers collect ammunition and pyrotechnics to activate them at parties”. The reliability was high (Cronbach's alpha 0.80).

#### Safety climate

The unit’s safety climate was evaluated by a questionnaire developed by Zohar and Luria evaluating the safety climate by examining the way the soldiers think the unit commanders relate to safety. The questionnaire included eight items, on a scale ranged between 1 (seldom) to 5 (very often). Examples of questions included: “When my commander is stressed he disregards the safety orders”. The inner reliability of the questionnaire was good (Cronbach's alpha was 0.79).

#### Personality evaluation

The Eysenek personality questionnaire revised short form (EPQ-R-S) was placed at the end of the research questionnaire [20]. Following the findings from a previous research, which demonstrated the significance of the dimensions P and L relating to risk taking these two dimensions were evaluated by 12 items for each dimension. The questionnaire used had known inner reliability and structure for which I have the normal level in the adult population in Israel.

### Procedure

The research period (2001–2002) was characterized by a top security alert; typical occurrences were encounters with terrorists, suicide bombings in the home front in Israel and at army bases. Approximately one third of the information was gathered in relatively peaceful regions at the time, mainly the Golan Heights, and the rest was gathered in regions that were highly threatened mainly in the Gaza strip and regions within the west bank.

## Results

Descriptive data of the research variables is presented in Table 1

**Table 1:** **Descriptive data of the study variables**

**L**	**Safety climate**	**Preparedness**	**Games with arms**	**GES**	**Perceived threat**	**Standard deviation**	**Mean**	
						0.83	0	Perceived threat
					0.02	0.10	2.05	GES
				-0.24**	0.04	0.72	1.56	Games with arms
			0.35**	0.02	0.17**	0.79	1.72	Exaggerated Preparedness
		-0.1*	-0.48**	0.35**	0.01	0.70	3.56	Safety climate
	0.13*	0.15**	0.08	0.14**	0.02	0.26	1.31	L
0.10*	-0.17**	0.05	0.17**	-0.19**	0.03	0.21	1.13	P

* = p<0.05, ** = p<0.01.

A positive correlation was found between exaggerated operational preparedness and games with arms (r = 0.35, p < 0.001) but as opposed to games with arms, exaggerated operational preparedness was only correlated with perceived operational threat (r = 0.17, p < 0.001). Since the correlation was low I continued to examine this variable on the unit level. I checked the mean of perceived threat in each unit and then divided the sample into 15 units with the highest levels of threat compared to 16 units with the lowest levels of threat. I performed a series of independent t-tests for each of the study’s variables and again I found a significant difference only in the variable of exaggerated operational preparedness, which partially supports the first research hypothesis: the mean of the ‘low threat’ group = 1.51 (n = 202, SD = 0.74) compared to the mean of the ‘high threat’ group = 1.91(n = 211, SD = 0.80). A statistically significant difference was found between the two groups (t (411) =5.1, p < 0.001).

Then the second research hypothesis was tested and indeed a correlation was found between General emotional state (GES) and games with small arms (r = −0.24, p < 0.01). Specifically, the higher the positive affect the lower the tendency to report dangerous games and vice-versa. The variable of emotional state was also correlated with safety climate, P and L dimensions. A further analysis used hierarchical regression, including categorical variable together with continuous variable. First I calculated a dummy variable which included the 16 units with low operational threat as first level and 15 units characterized by high operational threat as the second level. Then I introduced this variable in the first regression step together with the emotional state variable. Then I computed a new variable result from multiplying of these two variables. This variable was entered in the second step of the regression analysis. The results supported the second research hypothesis: I have found a main effect for General emotional state [β = −0.24, t = −4.87, p < 0.01] while operational threat and the interaction revealed no effect [β = −0.04, t = 0.9; β = −0.02, t = −0.1].

Next I tested the third research hypothesis that the correlation between general emotional state and dangerous games would be mediated by the safety climate. This analysis was also performed by hierarchal regression. As depicted in Table 2 a significant decline was found: the safety climate variable mediated the correlation between general emotional state and dangerous games. The implication is that even when a soldier is in a negative emotional state he doesn’t have a tendency for dangerous games, when his unit reinforces a high safety climate level.

**Table 2:** **Summary of hierarchical regression analysis for variables predicting risky games (N = 391)**

**Predicted variable-games with arms**			
*p*	*t*	*β*	
			*Step 1*
*.00*	*−5.00*	*-.24*	*GES*
			*Step 2*
*.08*	*−1.71*	*-.08*	*GES*
*.00*	*−9.50*	*-.45*	*Safety climate*

Step 1 Adj R^2^ = 0.06 (p < 0.01).

Step 2 Δ R^2^ = 0.18 (p < 0.01.

**Figure 1:**
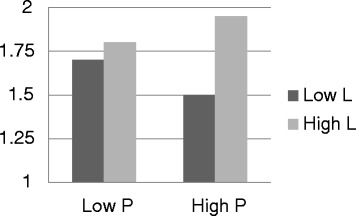
**Mean variable report of exaggerated preparedness for high level and low level in the dimensions (P) and (L) on the personality questionnaire EPQ-R-S.**

**Figure 2:**
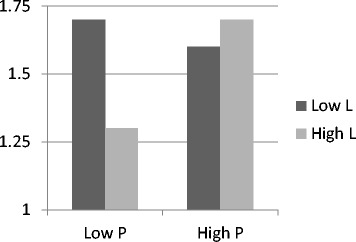
**Mean variable report of games with arms for high level and low level in the dimensions (P) and (L) on the personality questionnaire EPQ-R-S.**

Finally, I examined the fourth hypothesis regarding the correlation between the personality profile combined of the P and L dimensions and risk taking. First I performed multivariate analysis of Covariance (MACOVA) as following. The dimensions P and L were divided into two, according to the median in the reported population and were used as predictors. The impact of the safety climate and the perception of threat were neutralized by including them in the analysis as covariate variables. The predicted variables were two risky behaviors and they were tests simultaneously. Overall this analysis was found statistically significant [Hotelling’s T(2,383) = 3.8, p < 0.05]. Specifically, I hypothesized that individuals with a low level on the P dimension and a low level on the L dimension would be those to report a high tendency of risky behaviors. Then I tested the analysis with one dependent variable at a time. On the test of the impact of exaggerated preparedness I found a main effect to the variable L [F (1,383) = 13.1, p < 0.001] and statistical significant interaction of P and L [F (1,383) = 6.96, p < 0.01]. A statistical significant interaction of these two variables was also found on the variable games with arms [F (1,383) = 3.2, p < 0.1]. These findings are depicted in Figures 1 and 2. They demonstrate that the two dimensions P and L are correlated with taking unacceptable risks with arms. The nature of the interaction was different in each variable; possibly result from the unique nature of each behavior. This result warrants additional examination in the future.

## Discussion

This study examined the correlation between operational stress and taking unacceptable risks during operational deployment. The study reveals a phenomenon which is difficult to study in real time situations and often remains covert. The study focused on the fundamental characteristics of deployment: perception of the operational risk, general emotional state, and personality profile and examined them compared to two variables of unacceptable risky behaviors: exaggerated operational preparedness and games with arms and munitions. The unit safety climate was a possible mediator variable. The study hypotheses were partially confirmed. Limited evidence was found regarding a positive correlation between perception of the operational threat and exaggerated operational preparedness. The correlation found was low and it was not mediated by the safety climate. A support was found for hypothesis on the correlation between general emotional state and risky games. This correlation was mediated by safety climate in the unit. Finally, the personality profiles can partially explain the tendency to take unacceptable risks. It is important to note that firearms accidents are usually of high fatality rates. Their senseless characteristic has detrimental effects not only on the unit but on the bereavements of the victim's families [21]. Indeed great efforts were made to control this hazard – as in the case of other cases of unauthorized use of firearms especially concerning suicides [22].

### The correlation between operational stress, personality and risk taking

This study demonstrates that typical experiences of operational action can lead to unacceptable risk behaviors with arms and munitions and probably in other areas as driving. It appears each risky behavior has different causes: games with arms are correlated with general emotional state and low safety climate of a unit. While exaggerate operational preparedness is correlated with individual perception of operational threat and possibly – obviously not always substantiated- s sense control and mastery. The results repeat findings from the preliminary study, examining the correlation between the personality dimensions of the EPQ questionnaire and taking unacceptable risks with arms demonstrating that different combinations of the dimensions psychoticism (P) and lying (L) are correlated with taking these types of risks. Therefore I postulate that taking unacceptable risks typical of games with arms is an expression of hedonistic impulsivity and sensation seeking. I propose that continued research should use the term hypothesis ‘negative affect-games with arms’. While exaggerated operational preparedness reflects a dimension of ‘fight-flight’ response to anxiety. In future research of this topic I propose using the term 'hypothesis of threat-preparedness’; although the correlation between threat and exaggerated preparedness may not be linear.

The findings demonstrate that certain personality profiles tend to report a high degree of exaggerated preparedness. The appropriate profile in the present study and as opposed to the hypotheses is high dimensions of P and L. Possibly the willingness to take risks of this type are to some degree correlated with sense of independence, which allows certain individuals to freely take this form of action while they are in a perceived situation of threat. Possibly this profile is in some way correlated to a feeling of immunity or professional control which is researched in correlation to soldiers taking unnecessary risks when they return from operational action overseas.

Willingness to take risks during operational action is a complicated topic, since it can bring on the correct response to situations of danger and threat. Since operational action isolates the soldier from his surroundings and when he is confronted with risks he is alone and is required to utilize a degree of independence. However willingness for independent actions cannot be an excuse for irresponsibility or unprofessional conduct. Since taking excessive risks with arms can lead to a succession of faults resulting in accidents. The literature has differentiated between risks that are part of the mission and risks that are external to the mission and are not required. It appears professional combat leadership is a key element in the correct definition of this fine line and the ability to maintain it. According to reports in the literature military leadership can increase or decrease the tendency for taking unnecessary risks in training and there is no doubt that in the conditions of operational action when the soldier is away from the various sources of influence, direct leadership has an even greater impact. Safety climate is created from the way the soldier interprets his commander’s behavior regarding safety and all it entails. Therefore learning the characteristics of risk taking can reduce this risk. Accidents due to ‘excess motivation’ in production processes has been termed ‘compensatory behavior’ relating to operators of mechanical equipment that insist on operating technical or monitor equipment even when faulty, in order to achieve higher productivity and accomplishments.

## Conclusions

The consequences of unnecessary risk taking behavior with firearms and munitions are obvious. As recent study demonstrates the prevention of such hazard may result from very simple organizational procedures as limiting the access to firearms and munitions. It is essential the commanders will be aware of the exact nature of this risk. Low-level direct military leadership as well as stress control teams monitoring operational stress can use this research to explain the appropriate information and develop prevention activities in order to decrease the chances for accidents during deployment and on leave. Future research is needed on the differences between subgroups in the military: compulsory versus reserves or gender differences.
